# External cephalic version success rate and associated factors: Experience from a tertiary center in Sub-Saharan Africa: A cross-sectional study

**DOI:** 10.1371/journal.pone.0280404

**Published:** 2023-01-17

**Authors:** Abraham Fessehaye Sium, Wondimu Gudu, Tadesse Urgie, Gebeyehu Masresha

**Affiliations:** Department of Obstetrics and Gynecology, St. Paul’s Hospital Millennium Medical College, Addis Ababa, Ethiopia; University of Abuja Teaching Hospital, NIGERIA

## Abstract

**Objective:**

To determine the success rate of external cephalic version (ECV) and its associated factors in an Ethiopian setting.

**Material and methods:**

A total of 152 ECVs performed at the St. Paul’s Hospital Millennium Medical College, Addis Ababa, Ethiopia from June 1, 2018, up to March 30, 2019, were analyzed, using a prospective cross-sectional study design. Data were analyzed using SPSS version 21. Chi-square test of association was applied for categorical data analysis. Multivariate logistic regression analysis was used to determine predictors of success of ECV. Odds ratio, 95% CI, and P-value<0.05 were used to describe findings’ significance.

**Results:**

The success rate of ECV was 71.7%. ECV success rate did not differ between multiparous and nulliparous (AOR = 1.4, 95% CI 0.07–2.35), according to abdominal wall thickness status (AOR = 3.5, 95% Cl 0.29–42.40), and between unengaged and engaged presenting part (AOR = 1.1, 95% CI 0.26–4.74). A posterior placenta was associated with ECV success compared to anterior placenta (AOR = 1.14, 95% CI 1.03–2.60). Likewise, cases that experience no pain was associated with a higher ECV success rate (AOR 14.68, 95% CI 1.65–34.97). Soft uterine tone was also associated with a higher success rate compared to tense uterine tone (AOR = 3.89, 95% CI 0.02–0.39). Eighty-four percent of those mothers who had successful ECV had spontaneous vertex vaginal delivery.

**Conclusion:**

The success rate of ECV in this study is found to be 71.7%, which is higher than reports from previous studies. Absence of pain during the procedure, posterior placenta, and soft uterine tone were associated with successful ECV.

## 1. Introduction

Breech presentation complicates 3–4% of all pregnancies at term and many of these pregnancies are delivered by caesarean section [1, 2].External cephalic version (ECV) is an obstetric procedure that aims to rotate a breech fetus into the cephalic position by external maneuver through the maternal abdomen [3].

ECV decreases the likelihood that the fetus will be in a non-cephalic presentation at birth [4–6]. With a number needed to treat of three, it still remains a very efficient procedure to prevent a cesarean delivery [7]. There is not enough evidence from randomized trials to assess complications of ECV at term but large observational studies suggest that complications are rare [8].

External cephalic version (ECV), which is now routinely offered in developed countries, is not a popular procedure in the developing regions [9]. Overall, ECV is not widely practiced in East, Central, and Southern Africa, mainly owing to concerns related to safety and policy. Two studies from Sub-Saharan Africa demonstrate that only 21–52% of obstetricians practice ECV [10]. Ethiopia, the second largest population in Africa, with a large number of obstetricians in practice represents a wide range of obstetric practice among the Sub-Saharan Africa. The aim of this study was to determine the success rate of ECV and its associated factors in an Ethiopian setting.

## 2. Material and methods

Data for 152 ECV cases that were performed at the St. Paul’s Hospital Millennium Medical College (Addis Ababa, Ethiopia) from June1, 2018 to March 30, 2019were collected prospectively. St. Paul’s Hospital Millennium Medical College is one of the leading medical colleges in Ethiopia with various specialty and sub-specialty level patient care and training programs. Maternal-Fetal Medicine is one of the sub-specialty units at the department of obstetrics and gynecology of the college. Monthly, around 850–950 deliveries are attended at the hospital.

The study was conducted using a cross-sectional study design. Mothers with singleton pregnancy in breech presentation were recruited at ≥36 weeks of gestation during antenatal consultations. ECV procedures were provided by Maternal-Fetal-Medicine (MFM) specialist or MFM fellow (one physician per ECV client was assigned) at ultrasound room. All ECV providers were with at least 2 years of ECV performing experience. Exclusion criteria were the presence of placenta previa, placental abruption, oligohydramnios, fetal compromise, fetal death, severe fetal malformation, multiple gestation, Rhesus sensitization, uterine abnormalities, and clotting disorders.

On the day of the scheduled ECV, eligible participants had an ultrasound scan with the goals of estimating fetal weight, determining type of breech; placental location; and amount of amniotic fluid. All study participants received tocolytics and ECV was performed one hour after administration of a single dose oral administration of 20mg Nifedipin). None of the participants received any form of analgesia. The ECV procedure was carried out under ultrasound assessment, with the mother in a slight Trendelenburg position, and a ready C/S operating theatre for emergency interventions. Fetal heart rate monitoring was used to assess fetal well-being before and after the procedure. A change in presentation from non-cephalic to cephalic immediately after the ECV procedure was considered a successful ECV. Ultrasound was performed at the end of the procedure to confirm ECV success. Written informed consent was obtained from each study participant. A formal ethical clearance letter was obtained from St. Paul’s Hospital Millennium Medical College ethical board review (IRB).

Data on maternal age, maternal weight, gestational age, parity, amount of amniotic fluid, placental location, engagement status, abdominal wall status, and type of breech were collected on the same day immediately before performing ECV.

Engagement was defined as descent of at least 2/5. ECV related pain was scored out of 10 during the procedure (-no pain, 1-3-mild pain—feeling discomfort,4–6 –moderate—feels annoying, but tolerable, 7-10- severe—intolerable, kind of pain limits ones activity. Abdominal wall thickness and uterine tone were a subjective assessment made by respective ECV providers based on how easily they could feel fetal parts on abdominal examination. Engagement was defined as at least 1/5 fetal descent into the pelvis. Sonographic and procedural variables were collected during the procedure and immediately after the procedure. The ECV provider used both forward roll and backward roll techniques. It was standardized (all providers practiced it in the same manner). Amniotic fluid index was classified as low when AFI was 5-6cm; normal when AFI was 7-24cm and High when AFI was >25cm.Failed ECV was declared after a maximum of three attempts. Mode of delivery was reviewed from maternal charts. Data was collected by midwives and the principal investigator regularly supervised data consistency and completion

Data was entered into and analyzed using SPSS version 21. Chi-square test of association was done for categorical data analysis. Bivariate and multivariate logistic regression analysis was used to determine predictors of success of ECV. Odds ratio, 95% CI, and P-value were used to describe findings’ significance.

## 3. Results

A total of 152 case of ECV were recruited in this study. The overall success rate of ECV during the study period was 71.7% (109/152). There was no difference in socio-demographic characteristics distribution between those mothers who had successful ECV and those who had unsuccessful ECV (Table 1). In this study, 84% (92/152) of those mothers who had successful ECV had spontaneous vertex vaginal delivery (Table 2), compared to only 9.3%(4/43) in the unsuccessful ECV group, which is statistically significant finding (P-value = <0.001).

**Table 1:** Distribution of socio-demographic characteristics of study participants.

Variable	ECV Success status		Total P-value
	Successful n(%) n = 109	Unsuccessful n(%) n = 43	
**Age**			
<20	6(4.7)	3(6.9)	9(5.3) 0.397
20–25	33(30.8)	19(44.2)	52(34.1)
26–30	45(42.1)	16(37.2)	61(40.7)
31–35	17(15.9)	3(6.9)	20(13.3)
>35	8(6.5)	2(4.8)	10(7.2)
**Weight of the mother (kg)**			
≤65	62(56.8)	15(34.8)	77(50.7) 0.210
>65	47(43.2)	28(65.2)	75(49.3)
**Religion**			
Orthodox	58(53.3)	27(62.8)	85(56.0) 0.410
Protestant	19(17.8)	8(18.6)	27(17.7)
Muslim	32(28.9)	8(18.6)	40(26.3)
**Occupation**			
Unemployed	11(9.3)	2(4.6)	13(8.0) 0.426
Employed	17(15.9)	10(23.3)	27(17.8)
Farmer	5(4.7)	1(2.3)	6(4.0)
House wife	72(66.4)	30(69.8)	102(67.3)
Other	4(3.7)	-	4(2.7)
**Marital status**			
Married	108(99.1)	43(100.0)	152(99.3) 0.525
Divorced	1(0. 9)	-	1(0.7)

**Table 2:** Distribution of mode of delivery and fetal presentation at delivery.

Variable	ECV		P-value
	Successful n = 109	Unsuccessful n = 43	
**Mode of delivery**			
Vaginal delivery	92(84.4)	4(9.3)	0.000
Assisted breech delivery	3(2.8)	14(32.6)	
Cesarean delivery	14(12.8)	25(58.1)	

Vaginal delivery = cephalic vaginal delivery

On bivariate analysis (Table 3), for placental location, the highest success rate was obtained when the placenta was located in a posterior position (85%; 64/75, p-value = <0.001). Among the types of breech, the highest success rate was achieved for a frank breech (78.3%; 74/94, p-value = 0.02) as compared to non-frank breech (39.7%; 35/58).

**Table 3:** Bivariate analysis of predictors of successful ECV.

Variable	ECV		COR (95%C I)	P-value
	Successful(n = 109)	Unsuccessful n = 43		
**Maternal weight**				
≤65	64(79.5)	15(20.5)	1	
>65	45(62.2)	28(37.8)	2.35(1.12–4.91)	0.261
**Parity**				
Nulliparous	42(68.8)	19(31.2)	1	
Multiparous	67(73.6)	24(26.9)	1.02(0.39–1.67)	0.004
**Gestational age**				
36–37 week	30(65.2)	16(34.8)	1	
37–39 week	69(77.5)	20(22.5)	0.49(0.23–1.02)	0.059
>_40weeks	10(58.8)	7(41.2)		
**AFV**				
Low	8(70.0)	3(30.0)	1	
Normal	95(70.4)	39(29.5)	0.97(0.24–3.98)	0.976
High/	6(83.3)	1(16.7)	0.46(0.03–5.90)	0.556
**Placentation**				
Anterior	30(55.6)	24(44.4)	1	
Lateral	15(61.9)	8(38.1)	0.76(0.27–2.16)	0.618
Posterior	64(85.3)	11(14.7)	1.21(0.09–1.36)	0.000
**Descent of breech**				
Engaged	30(58.3)	20(41.7)	1	
Unengaged	79(77.5)	23(22.5)	0.41(0.19–0.85)	0.017
**Type of breech**				
Frank	74(78.3)	20(21.7)	2.36(1.14–4.87)	0.020
Non frank	35(60.3)	23(39.7)	1	
**Amniotic fluid**				
Low	17(63.0)	10(37.0)	1	
Normal	92(90.3)	10(9.7)	0.28(0.10–0.76)	0.130
**Fetal spine**				
Anterior	49(75.4)	16(24.6)	1	
Posterior	25(54.8)	19(45.2)	0.70(0.26–1.81)	0.463
Lateral	35(81.4)	8(18.6)	2.53(1.10–5.79)	0.028
**Abdominal wall**				
Unremarkable	76(72.4)	29(27.6)	1	
Thin	29(78.4)	8(21.6)	7.86(1.50–41.20)	0.015
Thick	4(25.0)	6(75.0)	0.72(0.29–1.76)	0.476
**Level of pain**				
no pain	19(90.5)	2(9.5)	18.04(3.48–93.6)	0.001
Mild pain	75(81.5)	17(18.5)	2.15(0.45–10.13)	0.332
Moderate pain	10(34.5)	19(65.5)	15.83(2.05–80.06)	0.008
Sever pain	5(37.5)	5(62.5)	1	
**Uterine Tone**				
Medium	16(48.5)	17(51.5)	1	
Soft	83(92.2)	7(7.8)	2.23(0.76–6.53)	0.141	0.141
Tense	10(29.6)	19(70.4)	0.07(0.02–0.22)	0.000
**Technique used**				
Forward roll	93(73.9)	32(26.0)	1	
Back flip	16(59.3)	11(40.7)	1.95(0.82–4.65)	0.130

Up on multiple logistic regression analysis (Table 4), ECV success rate did not differ between multiparous and nulliparous (AOR = 1.4, 95% CI 0.07–2.35), according to abdominal wall thickness status (AOR = 3.5, 95% Cl 0.29–42.40), and between engaged and unengaged presenting part (AOR = 1.1, 95% CI 0.26–4.74). A posterior placenta was associated with ECV success compared to anterior placenta (AOR = 1.14, 95% CI 1.03–2.60). Also, cases that experience no pain was associated with a higher ECV success rate (AOR 14.68, 95% CI 1.65–34.97). Tense uterine tone was associated with a lower success rate compared to soft uterine tone (AOR = 3.89, 95% CI 0.02–0.39).

**Table 4:** Multivariate logistic regression analysis of factors associated with success of ECV.

Variable	ECV		COR	AOR
	Successful N = 109	Unsuccessful n = 43		
**Parity**				
Nulliparous	42(68.8)	19(31.2)	1	
Multiparous	67(73.6)	24(26.9)	1.82(0.39–1.67)	1.41(0.07–2.35)
**Placentation**				
Anterior	30(55.6)	24(44.4)	1	
Lateral	15(61.9)	8(38.1)	0.76(0.27–2.16)	1.12(0.20–6.29)
Posterior	64(85.3)	11(14.7)	1.21(0.09–1.36)	1.14(1.03–2.60)
**Descent of breech**				
Engaged	30(58.3)	20(41.7)	1	
Unengaged	79(77.5)	23(22.5)	0.41(0.19–0.85)	1.10(0.26–4.74)
**Type of breech**				
Frank	74(78.3)	20(21.7)	2.36(1.14–4.87)	1.4(0.37–5.62)
Non frank	35(60.3)	23(39.7)	1	
**Fetal spine**				
Anterior	49(75.4)	16(24.6)	1	
Posterior	25(54.8)	19(45.2)	0.70(0.26–1.81)	0.63(0.13–3.06)
Lateral	35(81.4)	8(18.6)	2.53(1.10–5.79)	1.32(0.34–5.19)
**Abdominal wall**				
Unremarkable	76(72.4)	29(27.6)	1	
Thin	29(78.4)	8(21.6)	7.86(1.50–41.20)	3.54(0.29–42.40)
Thick	4(25.0)	6(75.0)	0.72(0.29–1.76)	0.28(0.05–1.38)
**Level of pain**				
no pain	19(90.5)	2(9.5)	18.04(3.48–20.60)	14.68(1.65–34.97)
Mild pain	75(81.5)	17(18.5)	2.15(0.45–10.13)	1.29(0.15–10.59)
Moderate pain	10(34.5)	19(65.5)	15.83(2.05–30.06)	9.74(0.61–35.12)
Sever pain	5(37.5)	5(62.5)	1	
**Uterine tone**				
Medium	16(48.5)	17(51.5)	1	
soft	83(92.2)	7(7.8)	2.23(0.76–6.53)	3.89(0.65–23.36)
Tense	10(29.6)	19(70.4)	0.07(0.02–0.22)	0.08(0.016–0.39)

## 4. Discussion

In this study, the success rate of ECV was 71.7% and 84% of those mothers who had successful ECV had spontaneous vertex vaginal delivery. The factors that were significantly associated with the success of ECV were: posterior placenta, absence of pain during the ECV procedure, and soft uterine tone.

According to data from the developed world, external cephalic version provides a means of reducing cesarean births, but an estimated 20–30% of eligible women are not being offered ECV [10]. However, in developing countries such as Sub-Saharan Africa, this practice is less commonly done. As stated earlier, two studies from Sub-Saharan Africa demonstrate that only 21–52% of obstetricians practice ECV in this region of Africa [11].

The goal of ECV is to increase the proportion of vertex presentations among fetuses that were formerly in the breech position near term [12]. According to the updated ACOG practice guidelines for ECV and based on a recent meta-analysis, the success rate of this procedure is reported to be 58% [13, 14]. Similarly, another report saz shows that the procedure results in a cephalic presentation in approximately 60% of the time [15]. The recent RCOG guideline recommends that women undergoing ECV should be informed that the success rate of ECV is approximately 50% [16].

In our study, from 152 ECV cases that were analyzed, 109(71.7%) had a successful ECV, which is higher than EVC success rates reported previously, from 47.2% in Netherlands [17], 58.5% in USA [18], 47% in UK [19], and 66.7% in Nigeria [20].

A possible explanation for this unique finding of higher ECV success rate in our study, compared to the reports from the fore-mentioned studies from around the world, could be difference in patient body habitus. Although, obesity among reproductive age group in Ethiopia is variable, it is lower than that reported from developed world. For example, the prevalence of overweight and obesity among reproductive age group in Addis Ababa, the city where at this study was conducted, is only 20.6% [21]. Morbid obesity has been found consistently to be associated with low ECV success rate in different studies [22].

In the literature, a variety of factors have been mentioned as predictors of ECV success. Earlier, Newman et al. found that parity, estimated fetal weight, breech station and placental implantation site were the most useful predictors of ECV success [23]. Likewise, J. Burgos et al found that a parity of 2 had a 3.74-times higher probability of ECV success than nulliparity (95% CI, 2.37–5.90) while a posterior placenta increased the success rate by 2.85 times compared with an anterior placenta (95% CI, 1.87–4.36), in his 2011 prospective analysis of 500 ECV maneuvers [24]. In the present study, experiencing no pain during the ECV procedure was associated with a higher ECV success rate (AOR 14.68, 95% CI 1.65–34.97). Similarly, posterior placenta was associated with ECV success compared to anterior placenta (AOR = 1.14, 95% CI 1.03–2.60). Soft uterine tone was also associated with a higher success rate compared to tense uterine tone (AOR = 3.89, 95% CI 0.02–0.39).

Most of the predictors of ECV success found in our study are consistent with reports from previous studies. For instance, Feyi-Waboso et al. found posterior placenta among the favorable factors for successful ECV [20]. Melo and colleagues in their cohort study of 18 years’ experience, demonstrated that low uterine tone is predictor of successful ECV success [19], which is consistent with our finding.

Women with a cephalic-presenting fetus at birth as a result of successful ECV are not at greater risk of obstetrical interventions at birth when compared with women with fetuses who spontaneously turn to a cephalic presentation in the third trimester [15]. In this study, 84% of those mothers who had successful ECV had spontaneous vertex vaginal delivery, which is comparable to the finding of 85.7% vaginal delivery rate in a previous similar study from UK [19].

Strengths of this study include; prospective data and Sub-Saharan Africa study setting, where there is scarcity of data on the practice of ECV. Possible selection bias in our study is the main limitation which flags for careful interpretation of this finding. The fact that clients who declined ECV procedure were not analyzed could have falsely elevated the success rate. The other limitations are the wide confidence interval represented in the key findings and the fact that neonatal outcomes were not included in the analysis. The operational definition of successful ECV- which should have been defined as cephalic presentation at birth, instead of only change into a cephalic position immediately after ECV procedure, is also another important limitation. There were 3 spontaneous reversions which ended up in having assisted vaginal breech delivery. We recommend a further analytic study with appropriate sample size allocation, inclusion of clients who decline ECV in the analysis, and the use of random sampling technique.

## 5. Conclusion

The success rate of ECV in this study is found to be 71.7%, which is higher than reports from previous studies. Absence of pain during procedure, posterior placenta, and soft uterine tone were the factors associated with ECV success. Although an interesting finding, possible selection bias might have contributed to this uniquely higher ECV success rate observed in our study.
